# Post-Contrast Acute Kidney Injury after Acute Stroke—Insights from a German Tertiary Care Center

**DOI:** 10.3390/jcm10235684

**Published:** 2021-12-02

**Authors:** Benedikt Frank, Jordi Kühne Escolà, Leoni Biermann-Ratjen, Anika Hüsing, Yan Li, Philipp Dammann, Ulrich Sure, Christoph Kleinschnitz, Michael Forsting, Martin Köhrmann, Cornelius Deuschl

**Affiliations:** 1 Center for Translational Neuro- and Behavioral Sciences (C-TNBS), Department of Neurology, University Hospital Essen, 45147 Essen, Germany; Benedikt.frank@uk-essen.de (B.F.); jordi.kuehneescola@uk-essen.de (J.K.E.); leonibiermannratjen@googlemail.com (L.B.-R.); Christoph.Kleinschnitz@uk-essen.de (C.K.); Martin.Koehrmann@uk-essen.de (M.K.); 2 Institute of Medical Informatics, Biometry and Epidemiology, University Hospital Essen, 45147 Essen, Germany; Anika.Huesing@uk-essen.de; 3 Institute of Diagnostic and Interventional Radiology and Neuroradiology, University Hospital Essen, 45147 Essen, Germany; Yan.Li@uk-essen.de (Y.L.); michael.forsting@uni-due.de (M.F.); 4 Department of Neurosurgery and Spine Surgery, University Hospital Essen, 45147 Essen, Germany; Philipp.Dammann@uk-essen.de (P.D.); Ulrich.Sure@uk-essen.de (U.S.)

**Keywords:** ischemic stroke, acute kidney injury, computed tomography angiography, computed tomography perfusion, contrast agent

## Abstract

Background: Our aim was to investigate the relationship between additional iodinated contrast medium (CM) application for acute stroke imaging and Post-Contrast Acute Kidney Injury (PC-AKI). Methods: We performed a retrospective analysis of consecutive patients with acute stroke who received a CT angiogram (CTA) with or without additional CT perfusion (CTP) at admission between 2017 and 2020. The primary endpoint was the incidence of PC-AKI. Potential causes of renal function impairment were recorded and logistic regression was performed to determine predictors of PC-AKI. Results: Of 3134 cases screened, *n* = 989 met the predefined inclusion criteria. PC-AKI occurred in 22 (5.4%) patients who received CTA only and 18 (3.1%) patients who received CTA and additional CTP (unadjusted OR, CI; 0.59, 0.29–1.05). In 31/40 (77.5%) patients who suffered PC-AKI, a non-CM-related cause of renal function impairment was identified. Stroke etiology (hemorrhagic vs. ischemic) and indicators of prior kidney disease were independent predictors of PC-AKI. Conclusions: Additional administration of CM for perfusion imaging in acute stroke did not show a relevant influence on the occurrence of PC-AKI. Patients with intracranial hemorrhage and/or prior kidney disease are at particular risk of developing AKI.

## 1. Introduction

Neuroradiological imaging is a key component to provide optimal clinical care for patients with acute stroke who are at high risk of disability or death. In the context of acute stroke assessment, a comprehensive CT protocol includes a combination of a non-contrast CT (NCCT) scan of the brain and a CT angiogram (CTA) of the extracranial and intracranial neck vessels to detect large vessel occlusion. Moreover, CT perfusion (CTP) is widely used in many tertiary neurovascular centers to identify patients suitable for reperfusion therapies in an extended time window, including endovascular treatment [1,2] as well as intravenous thrombolysis [3]. Apart from identifying the extent of infarct core and penumbral tissue, CTP provides important physiological information [4]. It may indicate the likelihood of progressive infarction, regardless of successful recanalization, and helps to identify patients requiring early control imaging for decisions on decompressive surgery. Each of the three components of multimodal CT provides unique diagnostic and prognostic information relevant to the management of acute ischemic stroke patients. Nevertheless, the debate regarding the implementation of routine CTP in CT stroke protocols is still ongoing, mainly due to additional iodinated contrast medium (CM) applications, radiation exposure, and time. To control and harmonize radiation exposure, different national and international guidelines were published to limit radiation exposure and state diagnostic reference levels [5,6]. Nevertheless, the additional use of iodinated contrast agents for CTP and the risk of contrast-associated acute kidney injury in these patients is an ongoing debate in daily clinical routine and needs further investigation [7,8]. In their latest guidelines, the Contrast Media Safety Committee (CMSC) of the European Society of Urogenital Radiology (ESUR) changed the term Contrast-Induced Nephropathy (CIN) to Post-Contrast Acute Kidney Injury (PC-AKI), emphasizing that assessment of creatinine increase after intravascular administration of contrast media is highly confounded by baseline parameters of patients [9]. Thus, PC-AKI is described as a correlative diagnosis, in which increments in plasma creatinine are mostly small and transient. Additional evidence from randomized controlled trials would be desirable; however, as these seem unfeasible due to ethical and practical constraints, data from large-scale observational studies are important.

We therefore aimed to examine the incidence, potential causes, and predictors of PC-AKI after acute stroke imaging in a large cohort of consecutive stroke patients in a tertiary care center in Germany.

## 2. Methods

### 2.1. Data Source and Study Population

This was a retrospective study, including data of all patients with acute stroke admitted to the comprehensive stroke center of the University Hospital Essen between January 2017 and March 2020 meeting the following inclusion criteria: acute ischemic or hemorrhagic stroke, age above 18 years, direct admission to our hospital (no secondary referrals or inhouse stroke), CTA and/or CTP study on the day of admission, and available creatinine tests at admission as well as during the following three days. Patients with prior chronic kidney disease requiring dialysis were excluded. All patients with acute ischemic stroke (AIS) and intracranial hemorrhage (ICH) admitted to our stroke center are prospectively collected in a local stroke registry database. The core dataset is entered by the attending physician on admission, completed during hospitalization, and validated by the attending senior stroke neurologist. Structured data of instrument-based examinations are entered by study personnel, and laboratory examinations are exported from the local archiving system. Finally, all data are validated by a senior stroke neurologist (BF). For patients who fulfilled the criteria of PC-AKI, potential causes of AKI other than CM administration were determined by reviewing hospital records.

This study was approved by the local ethics committee, and consent requirement was waived.

### 2.2. Imaging Protocol

A standardized Stroke-CT protocol for suspected stroke was used, including initial NCCT, CTA from aortic arch to vertex, and optional CTP of the brain performed as implemented by the vendor. All scans were performed at one of three commercially available, modern multi-slice CT scanners: one single-source 128-slice SOMATOM Definition AS+, one dual-source 128-slice SOMATOM Definition Flash, and one dual-source 192-slice SOMATOM Force (all Siemens Healthcare, Forchheim, Germany). For CTA, 70 mL of non-ionic iodinated contrast agent (Ultravist 300, Bayer, Leverkusen, Germany) was injected, and for CTP, an additional 50 mL of non-ionic iodinated contrast agent (Ultravist 300, Bayer) was injected. For mechanical thrombectomies, 100 mL of non-ionic iodinated contrast agent (Ultravist 300, Bayer) was initially available, followed by a refill of 50 mL if necessary.

### 2.3. Hydration Protocol

At our institution, stroke patients receive a total of 1000 mL intravenous volume supplementation with normal isotonic (0.9%) saline at high infusion rate (300 mL/h) at admission, followed by additional 1000 mL at 40 mL/h.

### 2.4. Statistical Analyses

The primary endpoint was Post-Contrast Acute Kidney Injury (PC-AKI), defined as an increase in serum creatinine of ≥0.3 mg/dL, or of ≥1.5–1.9 times baseline (KDIGO definition of AKI) in the 48–72 h following CM administration. We hereby used the definition of the updated ESUR Contrast Medium Safety Committee guidelines [9]. The secondary endpoint was the old concept of Contrast-Induced Nephropathy (CIN), defined as a relative increase in serum creatinine of >25% or 0.5 mg/dL [10].

We performed unadjusted and adjusted logistic regression to compare the occurrence of endpoints in stroke patients who received CTA on admission (CTA-only group) with those who received CTA and additional CTP (CTA-plus group). Additionally, in our primary analysis, the CTA-plus group included patients who received CM due to multiple CTA, CTP, or DSA for endovascular therapy (EVT). Results are presented as odds ratio (OR) and 95% confidence interval (95% CI), both for the effect of the CTA-group and the effect of different covariates.

Based on the nature and distribution of the data, descriptive statistics are presented as count and percent, median with interquartile range, or mean with standard deviation. Chi-square tests were used for categorical variables and, based on the distribution of data, t-tests, or Mann–Whitney–U tests were used for continuous variables. Statistical analyses were performed with SPSS (version 25.0, IBM Inc, Armond, NY, USA) and SAS (version 9.4, SAS Institute Inc., Cary, NC, USA), and a 2-sided *p* < 0.05 was considered to be the minimal level of statistical significance. The data that support the findings of this study are either shown in the manuscript or are available in the Supplementary Materials of this article.

## 3. Results

**Figure 1:**
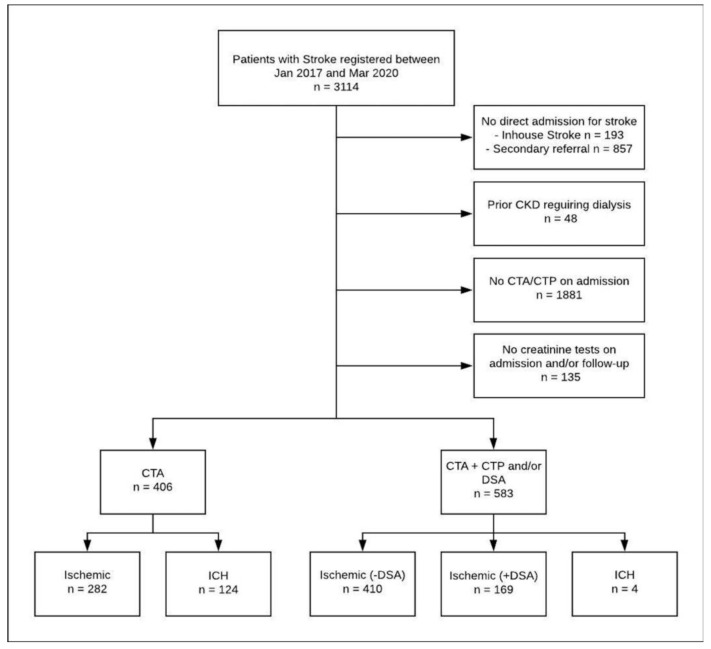
Flow chart with description of excluded patients and subgroups based on final discharge diagnosis and initial imaging method. CKD = Chronic Kidney Disease, CTA = Computed Tomography Angiography, CTP = Computed Tomography Perfusion, DSA = Digital Subtraction Angiography, ICH = Intracranial Hemorrhage.

**Figure 2:**
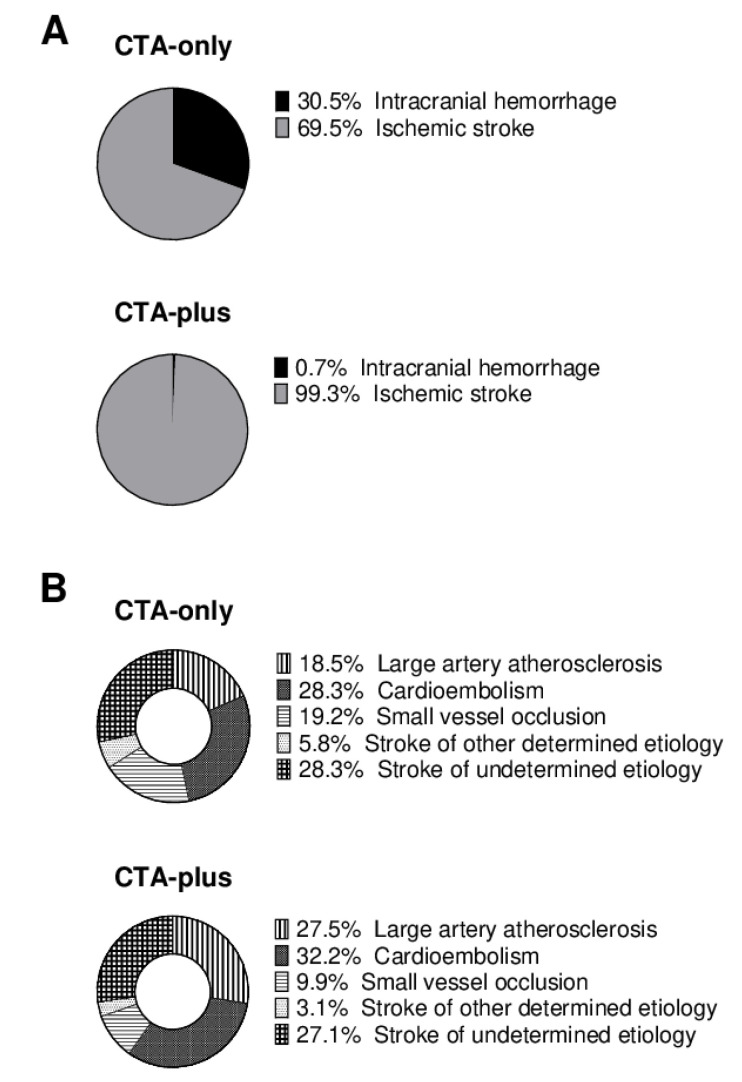
Visualization of stroke etiology in patients who received Computed Tomography Angiography only (CTA-only) or additional CT perfusion and/or Digital Subtraction Angiography (CTA-plus) on admission. (**A**) Relative proportion of intracranial hemorrhage vs. ischemic stroke (N = 989). (**B**) Etiology, according to the TOAST classification in patients with ischemic stroke (N = 861). Due to rounding, not all percentages add up to 100%.

A total of 3114 patients with acute ischemic or hemorrhagic stroke were registered between January 2017 and March 2020. A total of 989 patients met the predefined inclusion and exclusion criteria of the presented analysis (Figure 1), while 406 patients received CTA on admission (CTA-only group), 583 patients received CTA and additional CTP and/or DSA on admission (CTA-plus group). At baseline, patients in the CTA-plus group were significantly older, more often female, and more often had diabetes (Table 1). The distribution of stroke etiology is presented in Figure 2.

### 3.1. Incidence of Post-Contrast Acute Kidney Injury and Underlying Causes

An increase in creatinine corresponding to the criteria of PC-AKI occurred in 22 (5.4%) patients in the CTA-only group and 18 (3.1%) in the CTA-plus group (unadjusted OR 0.59, 0.29–1.05). CIN occurred in 27 (6.7%) patients in the CTA-only group and 22 (3.8%) in the CTA-plus group (unadjusted OR 0.60, 0.31–0.98). As no relevant differences between incidences of PC-AKI and CIN could be observed in further analysis, results on the latter are only reported in the Supplemental Materials.

In 31 of 40 cases that fulfilled the criteria of PC-AKI, common causes of AKI other than the administration of CM on admission were found to be a probable reason for creatinine increase: eight had severe infection or sepsis, another eight patients required intensive care with high amounts of catecholamine, six suffered from rhabdomyolysis and exsiccosis after lying on the floor prior to admission, three suffered from cardiogenic shock, two had cardiac decompensation with severe hypotonia, one received intravenous acyclovir despite chronic kidney failure, and another patient had post-renal AKI due to an obstructing tumor mass. The remaining nine stroke patients with PC-AKI had no other obvious reason for an increase in creatinine (CTA-only group *n* = 5, CTA-plus group *n* = 4). None of them required hemodialysis.

### 3.2. Predictors of Post-Contrast Acute Kidney Injury

Adjustment of logistic regression for single covariates (Supplemental Tables S1 and S2) revealed that ICH was the major risk factor for PC-AKI (OR for the covariate “stroke etiology” 0.25, 0.11–0.59). Adjusting for ICH nullified the group effect (OR 1.01; 0.45–2.27). While PC-AKI was more common in ICH (14 of 128; 10.9%) than in AIS (26 of 861; 3.0%), patients with ICH were overrepresented in the CTA-only group (30.5% vs. 0.7%).

The incidence of PC-AKI in AIS patients was 3.3% in the CTA-only group and 3.0% in the CTA-plus group (unadjusted OR 0.92, 95% CI 0.40–2.08). In patients with AIS, significant effects were observed for chronic kidney disease, elevated creatinine levels, reduced glomerular filtration rate, high NIHSS, and altered level of consciousness at admission when added as single covariates to the analysis of the CTA-group effect (Table 2 and Supplemental Table S3). Similar effects could be observed when looking at AIS patients without DSA on admission only (Supplemental Tables S4 and S5).

## 4. Discussion

We investigated PC-AKI in a large cohort of acute stroke patients, and our study reveals the following major findings: first, we did not observe any relationship between imaging modality at admission (CTA vs. CTA plus additional CTP and/or DSA) and incidence of PC-AKI. Second, non-CM-related causes of AKI were present in more than three-quarters of patients who fulfilled the criteria of PC-AKI following acute stroke imaging. Third, stroke etiology and indicators of prior kidney disease were major risk factors for the development of PC-AKI.

The overall incidence of PC-AKI was 4%, and cases were evenly distributed between imaging modality groups. The amount of CM administered was not a predicting variable for the incidence of PC-AKI, and none of the patients required hemodialysis. These findings are in line with previous studies that did not find an association between renal function impairment and CM-administration in acute stroke patients [10,11,12,13,14]. One meta-analysis even reported a lower incidence of AKI in patients with acute stroke who received CM on admission, as compared with patients who underwent NCCT [10]. However, this presumed advantage for patients receiving CM on admission is most likely due to a selection bias, as AIS patients not eligible for acute reperfusion therapy and thus only receiving NCCT on admission are more likely to have a worse outcome.

We found that up to 77.5% of patients with PC-AKI had a non-CM-related primary cause of renal function impairment in our study. This finding supports the hypothesis that the association of CM administration and AKI is coincident rather than causal and has probably been overestimated in the past [9].

Stroke etiology, however, was a major risk factor for PC-AKI in our cohort of acute stroke patients. While the rate of PC-AKI in patients with AIS was 3.0%, it was up to 10.9% in patients with ICH. These findings are in line with a meta-analysis that reported a general prevalence of AKI in 12.9% (95% CI 10.3–15.5) of patients with AIS, and 19.0% (95% CI 8.3–29.7) in patients with ICH [15]. The higher overall rates of AKI in this meta-analysis are not surprising, given that the definition of PC-AKI that was used in our study only considers the first 3 days after CM exposure. In general, patients with ICH are at particular risk for AKI, and this is likely due to a series of underlying mechanisms, including chronic hypertension and hypertensive kidney disease paired with antihypertensive treatment [16,17].

After excluding patients with ICH from the analysis in order to eliminate the masking effect and further investigate predictors of PC-AKI, the influence of other variables became apparent: indicators of prior kidney injury, as well as severity of the stroke index, were associated with the occurrence of PC-AKI after acute stroke imaging. 

We conducted a nonrandomized, retrospective analysis, and our study has limitations. Some potential factors not documented in our local stroke registry database, including prior or concomitant medication that may have contributed to PC-AKI, could have been missed. The amount of CM used for DSA was only documented in 50 mL steps. Despite our investigating a large cohort of acute stroke patients, the overall number of cases with a diagnosis of PC-AKI was relatively small. Furthermore, although a primary cause of renal impairment other than CM administration seemed evident from a clinical point of view in most patients with PC-AKI, our study cannot assess whether and to what extent CM administration may have been a precipitating factor for AKI in these patients.

Nonetheless, our analysis provides real-world data on PC-AKI after acute stroke imaging from a tertiary-care center with standardized stroke imaging and hydration protocols. In the absence of prospectively conducted randomized controlled trials, these findings give further insight into the controversial relationship between CM administration and AKI in acute stroke patients. 

## 5. Conclusions

In conclusion, the influence of additional CM administration for acute stroke imaging on the development of PC-AKI is minimal and most likely an innocent bystander. Stroke patients in general, and those with hemorrhagic stroke or prior kidney damage in particular, are at relevant risk of developing AKI. Hydration protocols and a high vigilance for the occurrence of AKI are of great importance in acute stroke patients.

## Figures and Tables

**Table 1:** Baseline information for *n* = 989 patients with acute stroke who received computed tomography angiography (CTA-only) on admission or additional CT perfusion and/or digital subtraction angiography (CTA-plus).

Characteristics	CTA-Only (*n* = 406)	CTA-Plus (*n* = 583)	*p*-Value
Age (years), mean (SD)	70.5 (13.6)	72.6 (13.6)	0.020
Sex: male	231 (56.9)	276/583 (47.3)	0.003
Hypertension	307 (75.6)	461 (79.1)	0.199
Diabetes	96 (23.6)	193 (33.1)	0.001
Chronic Kidney Disease	15 (3.7)	18 (3.1)	0.601
Atrial Fibrillation	120 (29.6)	196 (33.6)	0.243
Coronary Artery Disease	69 (17.0)	110 (18.9)	0.452
Peripheral Artery Disease	29 (7.1)	41 (7.0)	0.947
Premorbid Dependency ^a^	29 (7.4)	46 (7.9)	0.755
Tumor disease	71 (17.5)	90 (15.4)	0.088
Smoking	85 (20.9)	134 (23.0)	0.125
Pior Stroke	106 (26.1)	145 (24.9)	0.765
Prior TIA	13 (3.2)	22 (3.8)	0.338
NIHSS	6.0 (2.0–12.0)	7.0 (4.0–13.0)	0.001
Thrombolysis	85 (20.9)	332 (56.9)	<0.001
Time (h) from symptom onset	4.1 (1.2–17.1)	2.8 (1.1–9.3)	<0.001
Contrast Agent (mL)	70.0 (70.0–70.0)	120.0 (120.0–120.0)	<0.001
Creatinine (mg/dL)	1.1 (0.9–1.3)	1.1 (0.9–1.3)	0.300

Data are *n* (%) or median (IQR) if not indicated otherwise. Abbreviations: CTA = Computed Tomography Angiography; NIHSS, National Institutes of Health Stroke Scale; TIA. Data available in ^a^ *n* = 394 (CTA-only) and *n* = 582 (CTA-plus).

**Table 2:** Logistic regression for the occurrence of Post-Contrast Acute Kidney Injury (PC-AKI) in *n* = 692 ischemic stroke patients (covariate effect and group effect, respectively).

	Covariate Effect	Group Effect
	OR (95% CI)	OR (95% CI)
Sex, male vs. female	0.73 (0.33–1.61)	0.90 (0.39–2.05)
Age, per additional year	1.00 (0.97–1.03)	0.92 (0.40–2.08)
Chronic Kidney Disease, Yes vs. No	9.25 (3.18–26.9)	0.90 (0.39–2.07)
Premorbid Dependency, Yes vs. No	1.14 (0.26–4.96)	1.15 (0.47–2.81)
Diabetes, Yes vs. No	1.22 (0.54–2.79)	0.90 (0.40–2.06)
Hypertension, Yes vs. No	1.55 (0.53–4.55)	0.91 (0.40–2.07)
Coronary Artery Disease, Yes vs. No	2.26 (0.99–5.18)	0.93 (0.41–2.13)
NIHSS at Admission, per increment	1.05 (1.01–1.09)	0.80 (0.35–1.84)
Altered LOC at Admission, Yes vs. No	1.58 (1.00–2.49)	0.90 (0.40–2.05)
GFR < 30 mL/min, Yes vs. No §	7.47 (1.88–29.7)	1.14 (0.42–3.11)
Creatinine, per increment	1.72 (1.10–2.70)	0.96 (0.42–2.19)
Time from Onset to Admission, per increment	1.00 (1.00–1.00)	1.01 (0.43–2.38)
Atrial Fibrillation, Yes vs. No	0.73 (0.30–1.76)	0.92 (0.40–2.09)

OR, Odds ratio; CI, Confidence Interval; LOC, level of consciousness; NIHSS, National Institutes of Health Stroke Scale; GFR, Glomerular Filtration Rate. § in subset of 441 patients with available data.

## Supplementary Materials

The following are available online at https://www.mdpi.com/article/10.3390/jcm10235684/s1, Table S1: Logistic regression for the occurrence of Post-Contrast Acute Kidney Injury (PC-AKI) in all *n* = 989 stroke patients (covariate effect and group effect, respectively); Table S2: Logistic regression for the occurrence of Contrast-Induced Nephropathy (CIN) in all *n* = 989 stroke patients (covariate effect and group effect, respectively); Table S3: Logistic regression for the occurrence of Contrast-Induced Nephropathy (CIN) in *n* = 861 ischemic stroke patients (covariate effect and group effect, respectively); Table S4: Logistic regression for the occurrence of Post-Contrast Acute Kidney Injury (PC-AKI) in *n* = 692 ischemic stroke patients, excluding those receiving DSA (covariate effect and group effect, respectively); Table S5: Logistic regression for the occurrence of Contrast-Induced Nephropathy (CIN) in *n* = 692 ischemic stroke patients, excluding those receiving DSA (covariate effect and group effect, respectively).
