# Supplementary material for: Post-Contrast Acute Kidney Injury after Acute Stroke—Insights from a German Tertiary Care Center
Source: J Clin Med. 2021 Dec 2;10(23):5684. doi: 10.3390/jcm10235684 (PMC8658258; doi:10.3390/jcm10235684)
Supplement: Supplementary file 1 [file jcm-10-05684-s001.zip › jcm-1462184-supplementary.pdf]

Supplementary Materials

Supplemental Table S1. Logistic regression for the occurrence of Post-Contrast Acute Kidney Injury (PC-AKI) in all *n*=989 stroke patients (covariate effect and group effect, respectively).

	Covariate effect	Group effect
	OR (95% CI)	OR (95% CI)
Sex, male vs. female	1.11 (0.58 - 2.10)	0.56 (0.30 - 1.06)
Age, per additional year	0.98 (0.96 - 1.00)	0.58 (0.30 - 1.09)
Chronic Kidney Disease, Yes vs. No	5.94 (2.29 - 15.4)	0.56 (0.30 - 1.07)
Premorbid Dependency, Yes vs. No	0.66 (0.16 - 2.82)	0.60 (0.31 - 1.15)
Diabetes, Yes vs. No	1.11 (0.56 - 2.23)	0.55 (0.29 - 1.04)
Hypertension, Yes vs. No	1.71 (0.71 - 4.14)	0.55 (0.29 - 1.03)
Coronary Artery Disease, Yes vs. No	1.80 (0.88 - 3.69)	0.55 (0.29 - 1.04)
NIHSS at Admission, per increment	1.06 (1.03 - 1.09)	0.58 (0.31 - 1.12)
Altered LOC at Admission, Yes vs. No	1.84 (1.36 - 2.47)	0.66 (0.34 - 1.28)
GFR <30 ml/min, Yes vs. No	3.76 (1.01 - 14.0)	0.54 (0.24 - 1.18)
Creatinine, per increment	1.31 (0.90 - 1.91)	0.57 (0.30 - 1.08)
Time from Onset to Admission, per increment	1.00 (1.00 - 1.00)	0.57 (0.30 - 1.08)
Stroke Etiology, ischemic vs. hemorrhagic	0.25 (0.11 - 0.59)	1.01 (0.45 - 2.27)
Atrial Fibrillation, Yes vs. No	0.62 (0.29 - 1.33)	0.57 (0.30 - 1.07)

OR, Odds ratio; CI, Confidence Interval; LOC, level of consciousness; NIHSS, National Institutes of Health Stroke Scale; GFR, Glomerular Filtration Rate.
§ in subset of 517 patients with available data.

Supplemental Table S2. Logistic regression for the occurrence of Contrast-Induced Nephropathy (CIN) in all *n*=989 stroke patients (covariate effect and group effect, respectively).

	Covariate effect	Group effect
	OR (95% CI)	OR (95% CI)
Sex, male vs. female	0.78 (0.44 - 1.40)	0.54 (0.30 - 0.96)
Age, per additional year	0.99 (0.97 - 1.01)	0.57 (0.32 - 1.01)
Chronic Kidney Disease, Yes vs. No	4.65 (1.81 - 11.9)	0.56 (0.31 - 0.99)
Premorbid Dependency, Yes vs. No	0.25 (0.03 - 1.86)	0.58 (0.32 - 1.05)
Diabetes, Yes vs. No	0.93 (0.48 - 1.79)	0.55 (0.31 - 0.99)
Hypertension, Yes vs. No	1.02 (0.51 - 2.04)	0.55 (0.31 - 0.98)
Coronary Artery Disease, Yes vs. No	1.19 (0.58 - 2.44)	0.55 (0.31 - 0.98)
NIHSS at Admission, per increment	1.05 (1.03 - 1.08)	0.57 (0.32 - 1.02)
Altered LOC at Admission, Yes vs. No	1.81 (1.37 - 2.38)	0.65 (0.36 - 1.17)
GFR <30 ml/min, Yes vs. No §	2.06 (0.45 - 9.53)	0.48 (0.22 - 1.03)
Creatinine, per increment	0.80 (0.42 - 1.53)	0.55 (0.31 - 0.98)
Time from Onset to Admission, per increment	1.00 (1.00 - 1.00)	0.56 (0.31 - 1.00)
Stroke Etiology, ischemic vs. hemorrhagic	0.25 (0.12 - 0.55)	0.99 (0.48 - 2.06)
Atrial Fibrillation, Yes vs. No	0.62 (0.31 - 1.23)	0.56 (0.31 - 1.00)

OR, Odds ratio; CI, Confidence Interval; LOC, level of consciousness; NIHSS, National Institutes of Health Stroke Scale; GFR, Glomerular Filtration Rate.
§ in subset of 517 patients with available data.

Supplemental Table S3. Logistic regression for the occurrence of Contrast-Induced Nephropathy (CIN) in *n*=861 ischemic stroke patients (covariate effect and group effect, respectively).

	Covariate effect	Group effect
	OR (95% CI)	OR (95% CI)
Sex, male vs. female	0.44 (0.21 - 0.95)	0.88 (0.42 - 1.86)
Age, per additional year	1.01 (0.98 - 1.04)	0.92 (0.44 - 1.94)
Chronic Kidney Disease, Yes vs. No	7.14 (2.50 - 20.4)	0.91 (0.43 - 1.94)
Premorbid Dependency, Yes vs. No	0.42 (0.06 - 3.14)	1.11 (0.50 - 2.46)
Diabetes, Yes vs. No	1.04 (0.48 - 2.24)	0.92 (0.44 - 1.95)
Hypertension, Yes vs. No	0.99 (0.42 - 2.33)	0.93 (0.44 - 1.95)
Coronary Artery Disease, Yes vs. No	1.40 (0.62 - 3.18)	0.93 (0.44 - 1.96)
NIHSS at Admission, per increment	1.04 (1.01 - 1.08)	0.81 (0.38 - 1.73)
Altered LOC at Admission, Yes vs. No	1.68 (1.12 - 2.50)	0.91 (0.43 - 1.91)
GFR <30 ml/min, Yes vs. No §	4.67 (0.95 - 22.9)	1.31 (0.46 - 3.70)
Creatinine, per increment	1.05 (0.54 - 2.03)	0.94 (0.44 - 1.97)
Time from Onset to Admission, per increment	1.00 (1.00 - 1.00)	1.00 (0.46 - 2.16)
Atrial Fibrillation, Yes vs. No	0.78 (0.35 - 1.70)	0.93 (0.44 - 1.96)

OR, Odds ratio; CI, Confidence Interval; LOC, level of consciousness; NIHSS, National Institutes of Health Stroke Scale; GFR, Glomerular Filtration Rate.
§ in subset of 441 patients with available data.

Supplemental Table S4. Logistic regression for the occurrence of Post-Contrast Acute Kidney Injury (PC-AKI) in *n*=692 ischemic stroke patients, excluding those receiving DSA (covariate effect and group effect, respectively).

	Covariate effect	Group effect
	OR (95% CI)	OR (95% CI)
Sex, male vs. female	0.68 (0.27 - 1.72)	0.74 (0.30 - 1.85)
Age, per additional year	0.99 (0.96 - 1.03)	0.76 (0.31 - 1.91)
Chronic Kidney Disease, Yes vs. No	9.89 (2.97 - 32.9)	0.73 (0.29 - 1.84)
Premorbid Dependency, Yes vs. No	0.79 (0.10 - 6.06)	0.95 (0.36 - 2.54)
Diabetes, Yes vs. No	1.06 (0.39 - 2.84)	0.75 (0.30 - 1.89)
Hypertension, Yes vs. No	1.43 (0.41 - 4.97)	0.75 (0.30 - 1.87)
Coronary Artery Disease, Yes vs. No	3.13 (1.23 - 7.96)	0.78 (0.31 - 1.95)
NIHSS at Admission, per increment	1.05 (1.00 - 1.10)	0.75 (0.30 - 1.89)
Altered LOC at Admission, Yes vs. No	1.89 (1.12 - 3.19)	0.81 (0.32 - 2.04)
GFR <30 ml/min, Yes vs. No §	2.72 (0.32 - 23.3)	1.06 (0.35 - 3.24)
Creatinine, per increment	1.38 (0.75 - 2.52)	0.76 (0.31 - 1.91)
Time from Onset to Admission, per increment	1.00 (1.00 - 1.00)	0.84 (0.33 - 2.16)
Atrial Fibrillation, Yes vs. No	0.43 (0.12 - 1.50)	0.73 (0.29 - 1.83)

OR, Odds ratio; CI, Confidence Interval; LOC, level of consciousness; NIHSS, National Institutes of Health Stroke Scale; GFR, Glomerular Filtration Rate.
§ in subset of 346 patients with available data.

Supplemental Table S5. Logistic regression for the occurrence of Contrast-Induced Nephropathy (CIN) in *n*=692 ischemic stroke patients, excluding those receiving DSA (covariate effect and group effect, respectively).

	Covariate effect	Group effect
	OR (95% CI)	OR (95% CI)
Sex, male vs. female	0.38 (0.16 - 0.93)	0.76 (0.34 - 1.74)
Age, per additional year	1.00 (0.97 - 1.03)	0.81 (0.36 - 1.83)
Chronic Kidney Disease, Yes vs. No	7.32 (2.26 - 23.7)	0.78 (0.34 - 1.79)
Premorbid Dependency, Yes vs. No	n/a	0.97 (0.41 - 2.30)
Diabetes, Yes vs. No	0.93 (0.38 - 2.29)	0.81 (0.36 - 1.85)
Hypertension, Yes vs. No	0.78 (0.30 - 2.01)	0.82 (0.36 - 1.85)
Coronary Artery Disease, Yes vs. No	1.74 (0.71 - 4.29)	0.82 (0.36 - 1.85)
NIHSS at Admission, per increment	1.05 (1.01 - 1.10)	0.80 (0.35 - 1.82)
Altered LOC at Admission, Yes vs. No	1.88 (1.16 - 3.03)	0.86 (0.38 - 1.97)
GFR <30 ml/min, Yes vs. No §	2.91 (0.34 - 25.0)	1.47 (0.48 - 4.49)
Creatinine, per increment	0.66 (0.23 - 1.91)	0.80 (0.35 - 1.81)
Time from Onset to Admission, per increment	1.00 (1.00 - 1.00)	0.87 (0.38 - 2.02)
Atrial Fibrillation, Yes vs. No	0.46 (0.16 - 1.37)	0.78 (0.34 - 1.77)

OR, Odds ratio; CI, Confidence Interval; LOC, level of consciousness; n/a, not applicable; NIHSS, National Institutes of Health Stroke Scale; GFR, Glomerular Filtration Rate.

§ in subset of 346 patients with available data.
